# Linear mixed model to identify the relationship between grain yield and other yield related traits and genotype selection for sorghum

**DOI:** 10.1016/j.heliyon.2023.e17825

**Published:** 2023-07-01

**Authors:** Mulugeta Tesfa, Temesgen Zewotir, Solomon Assefa Derese, Denekew Bitew Belay, Hussein Shimelis

**Affiliations:** a Department of Statistics, College of Science, Bahir Dar University, P. O. Box, 79, Bahir Dar, Ethiopia; b Department of Statistics, College of Natural and Computational Sciences, Wollo University, P. O. Box, 1145, Dessie, Ethiopia; c School of Mathematics, Statistics & Computer Science, University of KwaZulu-Natal, Durban, 4041, South Africa; d Department of Plant Breeding, College of Agriculture, Woldia University, P. O. Box, 53, Woldia, Ethiopia; e School of Agricultural, Earth and Environmental Sciences, Africa Centre for Crop Improvement, College of Agriculture, Engineering & Science, University of KwaZulu-Natal, Pietermaritzburg, Durban, 4041, South Africa

**Keywords:** Fixed effects, Random effects, Principal component, Genotype selection, Best performer, Multivariate analysis

## Abstract

Sorghum is the most popular crop in arid and semi-arid areas, especially in Sub-Saharan African countries. Genotype effects, environmental and the interaction of genotype by environmental factors have an influence on phenotypic traits. The aim of the study is to identify the relationship between grain yield and other yield-related traits and select the genotypes which perform better in grain yield as well as to examine the association between the uncorrelated phenotypic traits and grain yield via mixed model. The data was generated using a lattice square design. Principal component analysis was used to generate uncorrelated variables for the mixed model. The study revealed that there was a difference in grain yield due to the treatment and there was a pairwise relationship among the phenotypic variables. 77.12% of the total variance of the original phenotypic variables was explained by the first three principal components and decided to use PCAs as input variables for the mixed model. All PCs had significant effects on grain yield as well as grain yield variability due to random effects associated with genotypes, genotype interaction by treatment, and replication within the treatment. The variability of grain yield due to genotype effect was explained about 45.73%, the variation of grain yield due to the interaction of genotype by the treatment was also explained about 39.06% and 1.55% of the variation of grain was explained by replication within treatment. The best performer genotypes recommended for mass production were G40 (Genotype 40), G186 (Genotype 186) and G196 (Genotype 196) without any constraint of environment. The genotypes recommended for mass production under irrigation conditions were G40 (Genotype 40), G62 (Genotype 62) and G192 (Genotype 192). G26 (Genotype 26), G55 (Genotype 55) and G49 (Genotype 49) were the genotypes recommended for mass production under stress conditions. Overall, the study recommends using a mixed model to fit the grain yield, and future work will focus on to evaluate the performance of genotypes under different environments and years of production.

## 1 Introduction

Sorghum (Sorghum bicolor (L.) Moench) is an important staple food for people in tropical arid and semi-arid regions of Africa, Asia and South America [[1], [2], [3]]. As a result, it is found to be the fifth most important cereal crop after wheat, maize, rice and barley [[4], [5], [6]]. It is also one of the most popular cereals in production around arid and semi-arid areas, especially in sub–Saharan African countries. It is the most important cereal crop in moisture-deficit areas of eastern Ethiopia and is widely distributed throughout the country. Out of the grain cereal crops, sorghum is a major food crop in Ethiopia and is high in land coverage and volume of production [7,8].

Phenotypic traits are the response of the genotypic effects, environmental and the interaction of genotype by environmental factors and the traits are measured using phenotypic characteristics which are multivariate data as they are used for the phenotypic traits and also correlated to each other. Correlation analysis is very important to measure the association between yield and some other component traits and also indicates collinearity among the predictors of the yield traits. When there are correlations, which are interdependence among the independent variables of the grain yield, between two independent variables that the values of the pairwise analysis [1,9].

A study conducted on sorghum variety performance in the lowlands of Wag and Lasta in Ethiopia and found that the yield had a direct and indirect relationship with pheno-agronomic traits and the result also indicates that there is a correlation between the pheno-agronomic traits [8].

Studies indicate that the phenotypic variables are correlated to each other and principal component analysis is a statistical tool to transform these correlated variables into uncorrelated (independent) new set of variables which maximizes the total variance of the phenotypic variables under the constraints being orthogonal to all preceding components [[10], [11], [12]]. The principal component analysis is important to screen out the correlation between phenotypic and genotype diversity, and it is very interesting to use as an input for further multivariate analysis techniques such as multivariate regression, clustering analysis and factor analysis [13,14].

Several studies applied multivariate data analysis such as principal component and cluster analysis to identify the correlation between the morphological traits and the genotype and the correlations among the phenotypic variables which found the presence of a strong positive correlation among the phenotypic characteristics such as head shape, midrib color, panicle exertion, glume color, presence of awns, grain color, glume cover and thousand seeds weight and strong negative correlation among days of flowering, leaf color, seed size, number of green leaves, grain color, thousand seed weight, awn presence, glume color, and inflorescence compactness [11,15,16].

The studies conducted by different investigators found that the yield was positive and significantly related to leaf length, leaf breadth and the number of leaves and indicated that these variables had a direct effect on yield. The study used principal component analysis to transform the morphological traits into a set of new traits having maximum variance with the constraints to be orthogonal to all preceding components, and cluster analysis was also used to classify the genotypes according to the Euclidian distance by ward's minimum variance linkage method [4,[17], [18], [19], [20]].

Studies noticed that the presence of association indicates that the traits are conditioned by the same set of genes in either positive or negative directions [8]. However, none of the studies mentioned the statistical models that quantify the relations between one phenotypic variable which is to be considered as a response variable, and the other phenotypic variables as well as covariates, considering these variables as predictor variables. Therefore, it is important to understand the linear or non-linear relationship between the grain yield and other agronomic and morphological traits of sorghum which provides insight to quantify important agronomic characteristics having difficulty measuring depending on easily measurable agronomic traits.

This study aims to identify the relationship between the sorghum yield and other yield-related traits using a model contains fixed and random effects and select the genotypes performing better using a mixed model examining the effect of yield related traits on grain yield.

This article is confined to 5 sections. The first section deals with an introduction to the study including a literature review and the aim of the study. Next, the second section describes the data and model formulations used for data analysis. The result of the study, which is presented in section 3, explains the main results of the study while the fourth section discusses and compares the results with other scientific works. Finally, the conclusion and the recommendation of the study are provided.

## 2 Data and methods

### 2.1 Experimental design and data collection

#### 2.1.1 Site description and experimental design

Genotypes were collected from the Ethiopian Biodiversity Institute (EBI). The institute was established in 1976 as a plant genetic resource center with a bilateral technical cooperation agreement between the Government of Ethiopia and Germany and in 1994 institute of Biodiversity conservation was established as well as nowadays its name is Ethiopian Biodiversity Institute (EBI) [21]. The institute is located in Addis Ababa, which is the capital of Ethiopia, with 9.0335206 latitudes and 38.7822931 longitudes. One hundred ninety-six genotypes were obtained from the institute that were used to identify the agronomic characteristics of sorghum in North East Ethiopia [20]. The experiment was held at the Kobo site of the Sirinka agriculture research center in 2014/2015 in Ethiopia. The site is located in the North Wollo zone in Amhara Regional state and far 40 km from Woldia, the center of the North Wollo Zone and 561 km far from Addis Ababa which is the capital of Ethiopia. The design of the experiment was an incomplete block design called lattice square design that contained 14 blocks, and each block contains 14 experimental units (plot) where the genotypes were applied and two replications for each treatment level that was a non-irrigated condition having a lack of water and the condition having sufficient [20,22]. The general layout of the design of the experiment is shown in Table 1 below.

**Table 1:** Lattice square design layout of the data.

Treatment → without irrigation (level 1)											
REP I						REP II					
	Genotype (196)						Genotype (196)				
Block ↓	1	2	3	⋯	14	Block ↓	1	2	3	⋯	14
1						1					
2						2					
3						3					
⋮						⋮					
14						14					
Treatment → With irrigation (level 2)											
REP I						REP II					
	Genotype (196)						Genotype (196)				
Block ↓	1	2	3	⋯	14	Block ↓	1	2	3	⋯	14
1						1					
2						2					
3						3					
⋮						⋮					
14						14					

#### 2.1.2 Data nature

The data collection procedure was planting 196 genotypes to identify sorghum genotypes capable of resisting the drought and genotypes having higher production of sorghum under the given treatment. From the experiment, 14 phenotypes characteristics of sorghum were measured under the experiment. In general, the counted phenotypic characteristics were the number of days of emerging (DE), days of flowering (DF), days of maturity (DM), the number of green leaves (NGL) and grain filling period (GFP) measured. However, panicle height (PH), panicle length (PL), panicle yield (PY), thousand seed weight (TSW), grain yield (GY), above-ground dry matter and harvest index (HI), which is the ratio of the grain yield and the above-ground dry matter, were the measured phenotypic characteristics of the experiment [23,24]. This study considered grain yield as a response variable and the input variables are treatment and principal components will be obtained from Principal component analysis considered as a fixed effect, and replication within treatment, genotype effect and the interaction of genotype by treatment are incorporated as random effects.

### 2.2 Statistical models

#### 2.2.1 Principal component analysis (PCA)

Principal component analysis (PCA), which transforms several correlated variables into several uncorrelated variables, is a multivariate technique for examining the relationships among several quantitative variables [10,15,25]. PCA is not an end by itself but it is an input for further multivariate analysis such as cluster analysis, factor analysis and multivariate regression [10]. Consider p set of correlated variables $X_{1},X_{2},X_{3}\ldots ,X_{p}$ with covariance matrix Σ and assume p set of variables $m_{1},m_{2},m_{3}\ldots ,m_{p}$ which are uncorrelated variables to each other and are linear combinations of the correlated variables, the principal component analysis has a form

$$\begin{array}{c} m_{1}=a_{1}^{\prime }X\\ \begin{array}{c} m_{2}=a_{2}^{\prime }X\\ \vdots \end{array}\\ m_{p}=a_{p}^{\prime }X \end{array}$$

The matrix notation of the principal component is

M=AX

Where

$$A=\left(\begin{array}{ccc} a_{11} & a_{12} & \begin{array}{cc} \cdots & a_{1p} \end{array}\\ \vdots & \vdots & \begin{array}{cc} \ddots & \vdots \end{array}\\ a_{1p} & a_{2p} & \begin{array}{cc} \ldots & a_{pp} \end{array} \end{array}\right);M=\left(\begin{array}{c} m_{1}\\ m_{2}\\ \begin{array}{c} \vdots \\ m_{p} \end{array} \end{array}\right);X=\left(\begin{array}{c} x_{1}\\ x_{2}\\ \begin{array}{c} \vdots \\ x_{p} \end{array} \end{array}\right)$$

The variance of $m_{i}$ is $a_{i}^{\prime }\Sigma a_{i}$ and covariance between $m_{i}$ and $m_{k}$ is $a_{i}^{\prime }\Sigma a_{k}$. Assume Σ has the eigenvalue-eigenvector pairs $\left(\lambda_{1},e_{1}\right),\left(\lambda_{2},e_{2}\right),\cdots ,\left(\lambda_{p},e_{p}\right)$ for all $\lambda_{i}\geq 0$; ∀i. The ith principal component is given by

$$m_{i}=\sum_{j=1}^{p}e_{ij}X_{j}=e_{i}^{\prime }X;i=1,2,\ldots ,p$$

Such that $var\left(m_{i}\right)=e_{i}^{\prime }\Sigma e_{i}=\lambda_{i}$ and $cov\left(m_{i},m_{k}\right)=e_{i}^{\prime }\Sigma e_{k}=0;\;i\neq k$. The sum of the variance of m is the sum of the eigenvalue of the covariance matrix and the proportion of the total variance due to the kth principal component is the kth eigenvalue divided by the total eigenvalue. The principal component, having the highest eigenvalue, has the highest variance proportion relatively [10,25].

#### 2.2.2 Linear mixed models

Linear mixed models are very flexible to fit the models which incorporate both fixed and random effects. Suppose that y is the response variable and consider the fixed effects which include the principal components ($m_{i};i=1,2,3$) and the treatment and the random effects including the replication within treatment, the genotype effect and the interaction of genotype by treatment [26]. The linear mixed model is given by Refs. [[27], [28], [29]]:

$$y_{ijkln}=\mu +\beta_{1}m_{1n}+\beta_{2}m_{2n}+\beta_{3}m_{3n}+\alpha_{i}+\rho_{j\left(i\right)}+\omega_{k}+{\left(\alpha \omega \right)}_{ik}+\varepsilon_{ijkln}$$

Where $y_{ijkln}$ is the outcome of the grain yield of the *n*th observation of *i*th treatment of *j*th replication and *l*th block of the *k*th genotype; μ is the overall mean; $\beta_{1},\beta_{2},and\;\beta_{3}$ are the coefficients for the 1st, 2nd and 3rd principal components which are considered to be fixed effects for the linear mixed model; $\alpha_{i}$ is the treatment effect which is a fixed effect; $\rho_{j\left(i\right)}\;\omega_{k},{\left(\alpha \omega \right)}_{ik}$ and $\varepsilon_{ijkln}$ are the random effects of replication within treatment, genotype, the interaction of treatment by genotype and the random error, respectively, and these are from a normal distribution with mean zero and variances $\sigma_{\rho \left(\alpha \right)}^{2},\sigma_{\omega }^{2},\sigma_{\alpha \omega }^{2}\;and\;\sigma_{\varepsilon }^{2}\text{,}$ respectively. The fixed effects and random effects are estimated using the concept of a best linear unbiased estimator (BLUE) and best linear unbiased predictor (BLUP) methods respectively, which both have the minimum variance among all linear unbiased estimators and minimum mean square error among all linear unbiased predictor respectively [12,27,30].

Linear mixed model parameters are Maximum Likelihood (ML), Restricted Maximum Likelihood (REML) and Minimum Norm Quadratic Unbiased Estimator (MINQUE) [31,32]. In this case, MINQUE is used to estimate the parameters of the random effects of the mixed model as the method does not require the normality assumption of the data [33]. The estimated values of the random effects help to estimate the parameters of the fixed effects of the mixed model by using generalized least squares (GLS) [34,35].

Model diagnosis of the linear mixed model is the most important visualization to assess the agreement between the model and the data which is evaluated by marginal and conditional residual plots to test normality, linearity, and homoscedasticity of the residuals and detect the outlier values. The other measure of diagnosis is the measure of influential diagnosis which helps to assess the extreme/outlier observation [32]. The analysis of this study was then performed using a mixed procedure of the SAS system (version 9.4) and R software (version R4.1.2).

## 3 Results of the study

### 3.1 Correlation coefficient analysis

The correlation analysis clearly shows the presence of positive and negative correlation among the phenotypic variables and also depicts the association between the grain yield and the other yield-related traits of sorghum.

**Fig. 1:**
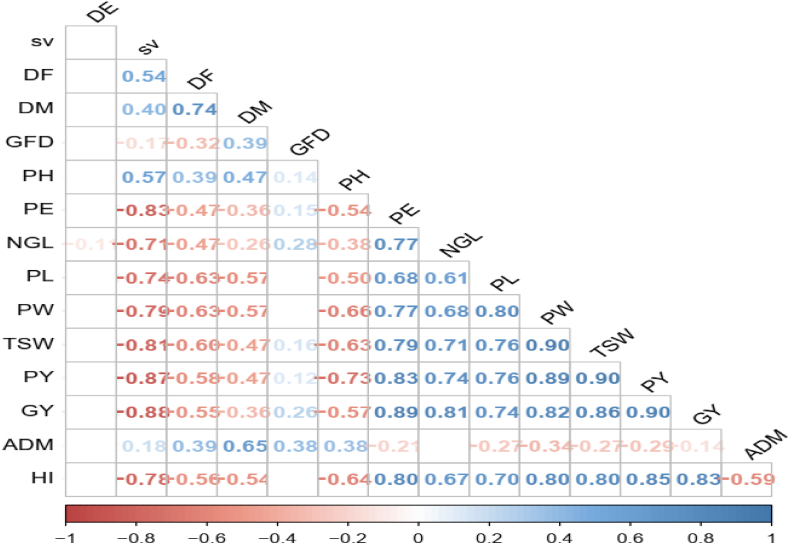
Pearson correlation coefficients.

Fig. 1 presents the results of the Pearson correlation coefficient which is used to test the null hypothesis that the population correlation coefficient is zero (there is no correlation coefficient among the variables). It shows the presence of a relationship among the phenotypic variables which displays the presence of a significant correlation between the grain yield and the possible phenotypic variables. The pairwise correlation indicates that there is a high correlation among the variables and explains the presence of multi-collinearity among the predictors such as the morphological variables (number of green leaves, panicle length, panicle width, panicle yield, harvest index) had a direct strong association pair wisely and the relationship among the phenology variables (seedling vigorous, days of emerging, days of flowering, days of maturity and grain filling period). The correlation between the grain yield and the morphological variables indicates the presence of a strong positive relationship except for panicle height which had an indirect strong association with the grain yield whereas the association between the grain yield and the phenological variables shows a negative relationship except for the grain filling period. The phenotypic variables which have a direct relationship with the grain yield were panicle exertion (r=0.89), number of green leaves (r=0.81), panicle length (r=0.74), panicle width (r=0.82), thousand seed weight (r=0.86) and panicle yield (r=0.90), and the phenotypic variables having an inverse relationship with grain yield were seedling vigorous (r=−0.88), days of flowering (r=−0.55), days of maturity (r=−0.36), panicle height (r=−0.57) and days of emerging (r=−0.08 (Pvalue<0.05). The pairwise correlation analysis indicates the presence of a relationship between the phenotypic variables and the grain yield. In addition, the presence of a strong correlation among the predictors indicates the precedence of multi-collinearity.

### 3.2 Principal component analysis

The result indicates the eigenvalues of the correlation matrix with the corresponding proportions of the components and the cumulative proportion of the components. 56.16% of the total variance was explained by the 1st principal component, and 13.9% of the total variance was explained by the 2nd component. 7.4% of the total variation was explained by the third principal component. The first three principal components had eigenvalues greater than 1 and explained more than three-fourths of the total variance. 77.46% of the total variance was explained by the first three principal components (Table 2). The first principal component had a larger positive association with the number of green leaves, panicle exertion, panicle length, panicle width, thousand seed weight and harvest index; however, it had a larger negative association with seedling vigorous and panicle height. Besides, these phenotypic characteristics explained 54.33% of the total variation of the phenotypic characteristics. The second principal component had days of maturity, grain filling period, number of green leaves and above-ground dry matter, and those variables explained 14.86% of the total variation of the phenotypic characteristics, which were more related to the 2nd principal component of the phenotypic characteristics of the sorghum production. The phenotypic variables such as days of emerging and days of flowering were related to the 3rd principal component which explain 7.92% of the total variance of phenotypic characteristics. The new variables which were transformed from the original phenotypic variables, and which are multivariate and correlated data, are uncorrelated and the dimension of the phenotypic variables was reduced into 3 sets of new variables.

**Table 2:** Eigenvalues and Eigenvectors of the correlation matrix.

Eigenvectors of the correlation matrix						
Unrotated				Rotated		
Variables	PCA1	PCA2	PCA3	PC1	PC2	PC3
Days of emerging (days)	−0.0248	−0.1104	**0.6942**	0.0012	−0.0020	**−0.0077**
Seedling vigorous (Scale)	**−0.3139**	−0.1534	−0.1342	**0.0243**	−0.0188	−0.0023
Days of flowering (days)	**−0.2551**	0.0455	**0.4711**	0.1629	**0.3669**	**−0.4004**
Days of maturity (days)	−0.2265	**0.4526**	0.2689	0.1507	**0.7024**	0.0963
Grain filling period (Days)	0.0307	**0.5769**	−0.2677	−0.0123	**0.3355**	**0.4967**
Number green Leave (#)	0.2731	0.2700	0.0177	−0.0673	**0.0859**	0.0695
Panicle height (cm)	−0.2534	0.1572	−0.2400	0.1906	0.0095	**0.6168**
Panicle exertion (cm)	0.3074	0.1594	0.1520	**−0.0725**	**0.0664**	0.0001
Panicle length (cm)	0.3013	0.0206	−0.0933	**−0.2060**	−0.1012	0.1719
Panicle width (cm)	0.3323	0.0096	0.0310	**−0.7321**	−0.0513	0.2457
Thousand seeds weight (gm)	0.3275	0.0972	0.0582	**−0.1690**	0.0797	0.0361
Panicle yield (gm)	0.3377	0.0838	0.1316	**−0.5389**	0.3908	−0.2892
Above-ground dry matter (t/ha)	−0.1525	0.5270	0.1282	0.0473	**0.2741**	0.1483
Harvest index (%)	0.3232	−0.0821	0.0800	**−0.0654**	−0.0067	−0.0391
Eigenvalue	7.8630	1.9456	1.0354			
Proportion	0.5616	0.139	0.074			
Cumulative	0.5616	0.7006	0.7746			

**Fig. 2:**
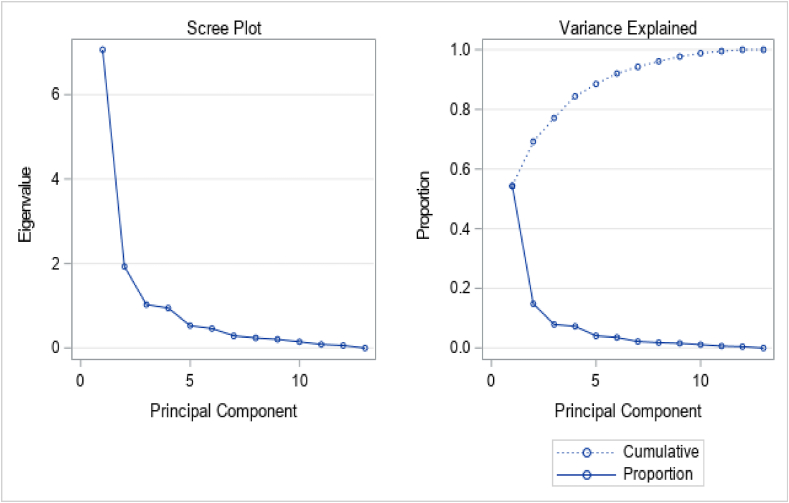
Principal component versus Eigen value (left) and the principal component versus the proportion (cumulative) (right).

Fig. 2 presents the principal component against the eigenvalue (left side of the plot) and the principal components against the proportion of the variances (cumulative) which shows an elbow after three principal components and the first three principal components contained approximately 78%, which is indicated by the dotted line of Fig. 2 (right hand side), of the total variance of the original variables. From this, we used three principal components which are important variables and input predictors (fixed effect for the grain yield) for the mixed model.

### 3.3 Results of mixed model

According to the result in Table 3, there was a linear association between the grain yield and the phenotypic components. The grain yield of sorghum has a direct relation with the first, second and third principal components of the phenotypic characteristics of sorghum having a positive estimate for the coefficient of the components and the *P*-values suggest to reject the null hypothesis that the effect of the components are significant on grain yield as Pvalue<0.05. A unit change of the first, second and third components of the phenotypic characteristics provided the increment of grain yield by 0.09841, 0.03223 and 0.01783 in log scale respectively. The average grain yields (in log scale) for irrigated and non-irrigated were 1.0197 and 0.9425 respectively. This shows that the presence of irrigation affects the mean difference in grain yield in the log scale compared to the absence of irrigation (non-irrigated). There was a significant difference in grain yield among the treatment (Pvalue<0.05). Random effect replication within treatment has an insignificant effect on the grain yield variability and 1.55% of the total variance of grain yield was associated with the variability of replication within treatment (Pvalue>0.05). The genotype effect has a significant effect on grain yield variability (Pvalue<0.05) and 45.73% of the variability of grain yield is associated with random effect genotype. The total variance of the grain yield, which is 39.73%, was related to the interaction of genotype by treatment and the effect of the random effect has a significant effect on grain yield variability. The variability of grain yield due to the block random effect was very small which causes to omit the block effect from the analysis.

**Table 3:** Tests on fixed and random effects significance for grain yield in log scale.

Type 3 Tests of Fixed Effects				
Fixed Effect	Numerator DF	Denominator DF	F Value	Pr > F
Treatment	1	779	36.91	<.0001
PCA1	1	779	2187.33	<.0001
PCA2	1	779	54.63	<.0001
PCA3	1	779	43.89	<.0001
Covariance Parameter Estimates				
Random effect	Estimate	Standard Error	Z Value	Pr > Z
Replication	0.00013	0.00010	1.3	0.0923
Genotype	0.003847	0.00063	6.12	<.0001
Treatment*Genotype	0.003286	0.00040	8.19	<.0001
Residual	0.001149	0.00009	13.3	<.0001
Solution for Fixed Effects				
Effect	Estimate	Standard Error	t Value (DF=779)	Pr > |t|
Intercept; μ	0.9425	0.01065	88.51	<.0001
Treatment (irrigated)	0.0772	0.01435	5.38	<.0001
Ref (non-irrigation)	0	.	.	.
PCA1; $\beta_{1}$	0.09841	0.001794	54.85	<.0001
PCA2; $\beta_{2}$	0.03223	0.003537	9.11	<.0001
PCA3; $\beta_{3}$	0.01783	0.002413	7.39	<.0001

PCA1 = 1st principal component, PCA2 = 2nd principal component, PCA3 = 3rd principal component.

The result displayed in Table 4 shows the top and least ten genotypes performance in grain yield. According to the result, the top three best-performer genotypes were G40 (Genotype 40), G186 (Genotype 186) and G196 (Genotype 196) with their BLUP estimates 0.1229, 0.1059 and 0.1016 respectively whereas G41 (Genotype 41), G66 (Genotype 66) and G136 (Genotype 136) were least three worst performer genotypes and their BLUP estimates of the genotypes were −0.1927, −0.1741 and −0.1391 respectively. The result indicates the standard error of the BLUP estimates was small (0.036).

**Table 4:** Summary of the BLUP estimates of the Genotypes performing better or worst.

Top ten best-performer genotypes					Least ten worst-performer genotypes				
Genotype	Estimate	Std Err Pred	t Value (*Df* = *779*)	Pr > |t|	Genotype	Estimate	Std Err Pred	t Value (*Df* = *779*)	Pr > |t|
40	0.1229	0.03665	3.35	0.0008	41	−0.1927	0.03645	−5.29	<.0001
186	0.1059	0.03657	2.9	0.0039	66	−0.1741	0.03629	−4.8	<.0001
196	0.1016	0.03716	2.73	0.0064	136	−0.1391	0.03656	−3.81	2E-04
187	0.09535	0.03615	2.64	0.0085	137	−0.1334	0.03646	−3.66	3E-04
93	0.09465	0.03645	2.6	0.0096	180	−0.1316	0.03623	−3.63	3E-04
116	0.08213	0.03627	2.26	0.0238	78	−0.128	0.03696	−3.46	6E-04
64	0.08118	0.03658	2.22	0.0267	39	−0.1265	0.03628	−3.49	5E-04
31	0.07453	0.03646	2.04	0.0413	108	−0.1067	0.03674	−2.91	0.004
92	0.07275	0.03625	2.01	0.0451	56	−0.1029	0.03639	−2.83	0.005
19	0.06932	0.03616	1.92	0.0556	85	−0.094	0.03633	−2.59	0.01

Std Err Pred = standard Error of Predictors; Df = Degree of freedom.

Table 5 presents the performance of the genotypes that coveys the BLUP estimates of the genotypes under irrigation conditions. This explained the genotypes which were appropriate genotypes for the wet environment. The result shows that G40 (Genotype 40), G62 (Genotype 62) and G192 (Genotype 192) were the top best performer genotypes under irrigated wet conditions and the BLUP estimates these genotypes were 0.1055, 0.09396 and 0.08959 respectively with relatively small standard error (0.038). On the other hand, the least three worst performer genotypes were G41 (Genotype 41), G55 (Genotype 55) and G2 (Genotype 2) with the corresponding BLUP estimates of −0.1505, −0.1219 and −0.1184 respectively.

**Table 5:** The performance of genotypes under irrigation.

Top ten best performer genotypes					Least ten worst performer genotypes				
Genotype	Estimate	Std Err Pred	t Value (*Df* = *779)*	Pr > |t|	Genotype	Estimate	Std Err Pred	t Value (*Df* = *779)*	Pr > |t|
40	0.1055	0.03792	2.78	0.0055	41	−0.1505	0.03797	−3.96	<.0001
62	0.09396	0.03785	2.48	0.0133	55	−0.1219	0.03805	−3.2	0.0014
192	0.08959	0.03782	2.37	0.0181	2	−0.1184	0.03786	−3.13	0.0018
104	0.08556	0.03786	2.26	0.0241	119	−0.1069	0.03789	−2.82	0.0049
157	0.08432	0.03793	2.22	0.0265	78	−0.1053	0.038	−2.77	0.0057
145	0.0816	0.03804	2.15	0.0322	36	−0.1001	0.03787	−2.64	0.0084
123	0.07913	0.03783	2.09	0.0368	39	−0.09966	0.03789	−2.63	0.0087
116	0.07713	0.03784	2.04	0.0418	180	−0.09445	0.03786	−2.49	0.0128
77	0.07669	0.03796	2.02	0.0437	53	−0.09213	0.03783	−2.44	0.0151
173	0.0763	0.03791	2.01	0.0445	8	−0.09204	0.03784	−2.43	0.0152

Table 6 indicates the best and worst performer genotypes under stress conditions. The result shows the top three best performer genotypes were G26 (Genotype 26), G55 (Genotype 55) and G49 (Genotype 49) and their estimates are 0.09087, 0.08751 and 0.085 with standard error 0.037. The least three worst performer genotypes are G66 (Genotype 66), G137 (Genotype 137) and G156 (Genotype 156) with their BLUP estimates of the three genotypes −0.1439, −0.107, −0.09075. As a result, Genotype 26, Genotype 55 and Genotype 49 are recommended for the mass production of sorghum under stress conditions whereas Genotype 66, Genotype 137 and Genotype 156 are not recommended for the mass production of sorghum under stress conditions.

**Table 6:** The performance of the genotype under stress condition.

Top ten best performer genotypes					Least ten worst performer genotypes				
Genotype	Estimate	Std Err Pred	t Value (*Df* = *779)*	Pr > |t|	Genotype	Estimate	Std Err Pred	t Value (*Df* = *779)*	Pr > |t|
26	0.09087	0.03793	2.4	0.0168	66	−0.1439	0.03783	−3.8	0.0002
55	0.08751	0.03799	2.3	0.0215	137	−0.107	0.03816	−2.8	0.0052
49	0.08568	0.03792	2.26	0.0241	156	−0.09075	0.03789	−2.4	0.0169
8	0.07853	0.03787	2.07	0.0384	157	−0.07438	0.03792	−1.96	0.0502
14	0.06403	0.03786	1.69	0.0912	145	−0.07191	0.03795	−1.9	0.0585
22	0.06394	0.03782	1.69	0.0913	192	−0.0681	0.03783	−1.8	0.0722
196	0.05946	0.03805	1.56	0.1185	189	−0.06637	0.03787	−1.75	0.0801
7	0.05917	0.03783	1.56	0.1183	195	−0.06536	0.0379	−1.72	0.085
1	0.05859	0.03792	1.55	0.1227	190	−0.05902	0.03793	−1.56	0.1201
12	0.05712	0.03782	1.51	0.1314	125	−0.05758	0.03784	−1.52	0.1285

## 4 Discussion of the study

The paper discussed and described the dimensional reduction of variables and produced an uncorrelated new set of variables, which used the principal component variables as an input to estimate the response variable, in this case, grain yield, for the linear mixed model which is a very flexible way of identifying the relationship between the response variable and predictor variables. The results of the descriptive statistics indicate the difference in grain yield of the treatment (irrigated and non-irrigated) and the observation of grain yield under irrigated conditions is more consistent than the observation of grain yield under non-irrigated conditions. This result is consistent with other studies which were investigated the impact of drought on sorghum production and the effect of the genotypes on sorghum production [20,36,37].

The correlations among the phenotypic variables are significant which indicates the presence of association among the phenotypic variables and an indication of developing a statistical linear relationship between one trait, in this case, grain yield, and other related traits. This study agreed with the studies which indicated the relationship between grain yield and other yield-related traits [14,17,38].

The principal component analysis is the most important multivariate data analysis tool which helps to generate uncorrelated variables from correlated variables by maximizing the variance of original variables. PCA1 explained 54.33% of the total variance of the phenotypic traits, and PCA2 also explained 14.86% of the total variance of the phenotypic traits such that PCA1 and PCA2 are uncorrelated (independent) to each other. PCA3, which is uncorrelated to the other preceding two PCAs, explained 7.92% of the total variance of the phenotypic variable. The first three principal components explained 77.12% of the total variance of the phenotypic variables [13,20,38]. The principal component analysis is mainly important to find independent variables from correlated variables and essential to data reduction for high dimensional data. These new independent variables were used as input variables for statistical models to model the response variable. A study was conducted by other investigators who used the principal components as independent variables for linear models [12].

The result of the linear mixed model presented the quantitative measure of grain yield against the fixed effects namely the treatment effect (irrigation), the principal components (PCA1, PCA2 and PCA3) and the random effects, that were used in a linear mixed model, are replication within irrigation, genotype and the interaction of genotype and treatment [36]. The fixed effects were statistically significant on grain yield. The grain yield increased due to principal components as there were a linear combination of the other yield related traits and these showed the presence of a direct relationship between grain yield and other yield related traits. The variability of grain yield was significantly associated with the effect of genotype, the interaction of genotype by treatment and the replication within treatment. The genotypes may generate different production under several environmental conditions, for instance, a genotype that can produce high yield under irrigation, may not produce better yield under stress conditions that is due to the presence of different capacities in drought resistance among genotypes [36,37]. The result of the random effects showed that G40 (Genotype 40), G186 (Genotype 186) and G196 (Genotype 196) were the best performer genotypes without any constraint of the environmental conditions and the result recommends these genotypes for the mass production in Ethiopia. This agreed with the study in the same region [20,36]. Moreover, the performance of genotypes is different under the treatment levels. The top three best-performer genotypes under irrigation (sufficient water supply) were G40 (Genotype 40), G62 (Genotype 62) and G192 (Genotype 192) which the study recommends for mass production in irrigated regions. The recommended genotypes for mass production under stress (insufficient water supply) are G26 (Genotype 26), G55 (Genotype 55) and G49 (Genotype 49). These genotypes were found to resist the impact of drought which was revealed by the shortage of water during the growth of the genotypes [20].

## 5 Conclusion

The study indicates that the effect of treatment was on grain yield and also shows the presence of a pairwise relationship among the phenotypic variables, particularly the correlation coefficient between grain yield and the other phenotypic variables that have statistically significant association. Based on the correlation analysis, the phenotypic variables were correlated and principal component analysis was used to generate new uncorrelated (independent) phenotypic variables. The principal component analysis shows that 77.12% of the total variance of the original variables was explained by the three principal components. The outcomes of the principal component analysis were used as independent variables (fixed effects) for linear mixed model. The production of grain yield under irrigation is relatively high compared with the production of the grain yield under stress condition that leads to concluded the impact of drought on grain yield. The effect of principal components was important to estimate the grain yield in which all principal components had a direct linear relationship to the grain yield as the coefficients of the components are positive. The variability of grain yield due to random effects was associated with replication within treatment, genotypes and the interaction of genotype by treatment. According to the proportion of the total variation of the grain yield, the genotype and the interaction of genotype by treatment have high contribution on the variability of grain yield with respect to their contribution as well as the contribution of the replication within the treatments in grain yield is very small relative to other random effects. The best performer genotypes recommended for mass production were G40 (Genotype 40), G186 (Genotype 186) and G196 (Genotype 196) without any constraint of environment. Under irrigated condition, G40 (Genotype 40), G62 (Genotype 62) and G192 (Genotype 192) are recommended genotypes for mass production. On the other hand, in stress condition, G26 (Genotype 26), G55 (Genotype 55) and G49 (Genotype 49) are the genotypes recommended for mass production. Overall, the study recommended the use of the linear mixed model to fit grain yield against various correlated phenotypic characteristics. The future work will focus on to evaluate the performance of genotypes under different environments (location) and years of production.

## Author contribution statement

Mulugeta Tesfa: Temesgen Zewotir: Denekew Bitew Belay: Analyzed and interpreted the data; Wrote the paper.

Solomon Assefa Derese: Conceived and designed the experiments; Performed the experiments; Contributed reagents, materials, analysis tools or data.

Hussien Shimelis: Conceived and designed the experiments; Contributed reagents, materials, analysis tools or data.

## Data availability statement

Data included in article/supp. Material/referenced in article.

## Additional information

No additional information is available for this paper.

## Declaration of competing interest

We have no known competing financial interest or personal relationships.
